# Physiologically-based testing system for the mechanical characterization of prosthetic vein valves

**DOI:** 10.1186/1475-925X-6-29

**Published:** 2007-07-13

**Authors:** Stanley E Rittgers, Matt T Oberdier, Sharath Pottala

**Affiliations:** 1 Department of Biomedical Engineering, The University of Akron, Akron, OH 44325, USA

## Abstract

Due to the relatively limited amount of work done to date on developing prosthetic vein (as opposed to cardiac) valves, advances in this topic require progress in three distinct areas: 1) improved device design, 2) relevant device testing conditions, and, 3) appropriate parameters for evaluation of results. It is the purpose of this paper to address two of these issues (#2 and #3) by: 1) performing a study of normal volunteers to quantify the anatomy and hemodynamic features of healthy venous valves, 2) construction of a 2-step, *in vitro *testing procedure, which simulates both physiologic and postural conditions seen in the lower extremity venous system, and, 3) defining several modified and new parameters which quantify dynamic valve characteristics.

## Background

Although over 50 designs for prosthetic cardiac valves have been put forward over the past 40+ years and extensive testing performed on many of these devices resulting in millions of valves being implanted in patients [1], only a handful of concepts have been put forward for a prosthetic venous valve [2-7] and only 1 or two devices have reached clinical trials as yet [8]. Simultaneously, it is estimated that between 6–7 million patients suffer from chronic venous insufficiency [CVI] of the legs with approximately 900,000 new patients be diagnosed annually in the United States [9]. Of these, about 1–2% will develop venous stasis ulcers of the lower extremities with many of them potentially benefiting from a satisfactory venous implant.

Thus, there remains a large, unmet demand for a functioning prosthetic vein valve, particularly one designed for implant into the proximal veins. The overall objectives of this study, then, were to create a prosthetic vein valve capable of successful clinical implantation which would: 1) provide minimal resistance to antegrade flow, 2) re-establish prevention of reflux flow, and, 3) remain patent for a minimum time period. To accomplish this, several novel designs of a valve frame and leaflet component were conceived which then required evaluation under relevant venous conditions. Thus, *in vitro *testing systems were developed to provide physiologic conditions under typical postural positions appropriate for a valve in the common femoral vein (CFV) position. Measurements were made of standard dynamic variables and both traditional and novel parameters were also derived from these data.

Previous related work was done by Hasaniya who used a rudimentary system to evaluate a vein valve constructed out of bovine jugular vein [7]. His approach was to simply place the construct vertically into a closed dynamic flow system such that the leaflet operation could be observed under ultrasound imaging. The valve system was "evaluated continuously at various pressures", including 200–260 mmHg.

One of the more detailed systems was described by Qui et al. [10] who evaluated the fluid dynamics of two venous valve models made from bovine jugular vein valves. The system had the ability to control the steady forward flow through a horizontally mounted, 25 mm ID valve test section by adjusting the height of a downstream reservoir (up to 20 cm) relative to that of an upstream reservoir (fixed at 30 cm). Oscillatory effects were introduced by a third tank placed downstream and mounted on a rotary wheel such that it induced an additional backpressure from 0 to 13.5 cm H_2_O. This feature was designed to simulate respiratory and/or muscle pump actions. Flow was measured using an EMF, pressure was monitored at a single point immediately downstream of the valve and a glycerin in water blood analog fluid was used. Valve opening area was measured using the electric current generated by a photo detector that sensed the amount of light intensity from a He-Ne laser beam expanded to 15 mm diameter that was able to pass through the valve along its axis. Axial velocities upstream of the valve, at the valve exit, and 2 cm downstream of the valve exit were measured with a 4 W Argon-ion laser Doppler anemometer. This system was used to test a stented, formaldehyde treated bovine jugular vein valve and a stentless bovine jugular vein valve conduit. Their results confirmed the theory that vein valves are operated by a pressure differential rather than flow and that no reflux is needed to completely close the valve. Additionally, it was shown that the vein valve sinus expands rapidly upon backpressure, which was deemed to be a critical characteristic to consider when designing prosthetic venous valves.

A number of other studies have been performed where compliant tubing was used to contain the valve [11-14]. In particular, Raju et al. [15,16] showed that a collapsible tube helps to buffer pressure variations in the vein and, upon closure of the valve, the tube below the valve collapses, producing venous pressure reduction. More recently, Buescher et al. [17] tested thin-walled latex and polyurethane film vein valves placed in a 12.7 mm latex tube which was mounted vertically and exposed to flow moving in either the antegrade or retrograde directions (depending upon input/output connections) between an arterial and a venous reservoir. A fluid medium (either 0.9% saline or 42 wt% glycerin solution) was gradually drained from the arterial reservoir into the venous reservoir over a height variation that produced flow rates from 0 to 15 ml/s. Measurements were made of the mass flow rate, various pressures including the differential pressure across the valve, and cross-sectional images from an arthroscopic camera. The primary output variable analyzed was the competency, C, which was defined as the percent ratio of the antegrade minus the retrograde flow rate divided by the antegrade flow rate to evaluate the effects of leaflet gap widths and sinus sizes.

## Methods

A study was first performed on normal volunteers to quantify the anatomy and dynamics of healthy venous valves (The University of Akron Institutional Review Board, Application #20040140). A total of eleven superficial (SFV) and common femoral veins (CFV) in eight individuals were non-invasively examined using a Duplex ultrasound scanner (Siemens Medical System Incorporated, Ultrasound Group, Issaquah, WA). Because venous dynamics are a function of both hydrostatic and transmural pressures, measurements were taken under a combination of postural positions and physiologic conditions. For example, venous pressures significantly increase by: 1) elevating from a supine to a standing position, 2) inducing gait via the activation of the skeletal muscle pump, and, 3) breathing through changes in intrathoracic pressure. Thus, measurements were taken under various postural and dynamic conditions, including supine breathing, supine ankle flexion, standing breathing, and standing ankle flexion. Measurements were also taken during a Valsalva maneuver, which is defined as an attempt to forcibly exhale while keeping the glottis closed. This is an extreme condition in which the thoracic vena cava collapses leading to the application of a high retrograde pressure to the most proximal venous valves. Parameters obtained using B-mode imaging included CFV diameter, presence or absence of valve sinuses, SFV diameter, leaflet length, and SFV leaflet to wall angle. Peak SFV augmentation velocity and cycle period were obtained from the single channel pulse Doppler mode. Other parameters were derived from the raw data and included: leaflet to SFV diameter ratio, SFV area, SFV flow rate, SFV peak Reynolds number, and SFV Womersley number. Although these data are too extensive to report here, the average values were used as a guide for designing an *in vitro *flow test system and the first generation of prototypic, prosthetic valves.

The next step in the development process was to identify device designs, which minimally satisfied the criteria of 1) opening upon antegrade flow and 2) closing upon retrograde flow. Once conceived, these designs were then modified by varying several key dimensional parameters in order to provide a range of configurations that could result in measurable differences in valve function. [Note: A detailed description of these devices and their functional outcomes will be presented in a subsequent publication.] Furthermore, it was decided that a 2-stage testing process would provide the ability to first observe the devices in a more global operation (i.e. opening and closing, breakage, etc.) before proceeding with more rigorous testing. Thus, two test systems were built, one of which provided first-cut information about the valve's responsiveness to basic flow conditions (forward steady flow and step reverse flow) and the second of which provided more realistic physiologic conditions. Because of the rough, observational nature of the first testing protocol, the devices and test system were scaled up by a factor of 2:1 in order to better observe device operation. Those devices which passed this vertical column Mock-up Steady Flow System testing were then re-fabricated as 1:1 scale devices and more thoroughly evaluated in the horizontal Physiologic Flow System.

### a. 2:1 Mock-Up System

**Figure 1:**
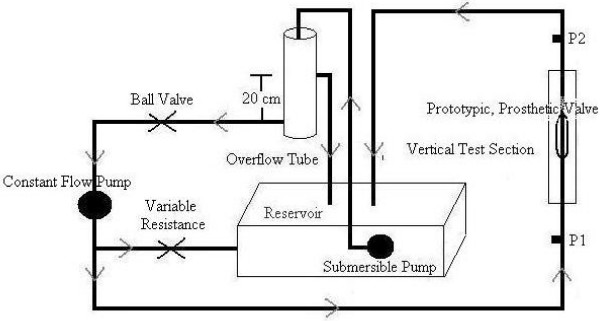
A schematic of the vertical column 2:1 Mock-Up System.

The main purpose of the 2:1 Mock-Up System was to mimic conditions in the standing position where demands on the valve were most extreme. Therefore, a vertical column was constructed in which the candidate devices were placed (Fig. 1). The outlet of the column was at a height of 55 cm above the test specimen to simulate the elevation of the heart above a proximal vein valve in the standing position. For simplicity, a constant flow pump (IWAKI PULSA Feeder, Interpace Corporation, Rochester, NY) was used to provide the driving pressure and tap water was used as the test fluid. Initially, valve misalignment within the test section and system leakage at the valve connection were commonly experienced. Both of these conditions were corrected through the use of a threaded adaptor to secure the valve Mock-ups in place. Matching threading was added to each of the prototype valves, which could then be screwed into the system after the application of Teflon^® ^tape. The flow loop was closed by lowering a one-inch inner diameter Tygon^® ^tube over the valve and securing it with a clamp. Upstream of the test section was a reservoir with a submersible pump (Model 1, Little Giant Pump Company, Oklahoma City, OK) that filled a constant elevation (20 cm) reservoir (Tower I) that, in turn, functioned to prime the constant flow pump. The output of the constant flow pump to the test section was regulated by a variable resistor placed in a recycle stream to the reservoir. This allowed the flow rate delivered to the test section to be set without altering the pump output.

Upon scaling up to the 2:1 Mock-up system, a balance of viscous and inertial forces was maintained by matching the Reynolds numbers of the system to that obtained in the Normal Volunteer Study:

Re⁡=ρNLvNLDNLμNL=ρMUvMUDMUμMU
 MathType@MTEF@5@5@+=feaafiart1ev1aaatCvAUfKttLearuWrP9MDH5MBPbIqV92AaeXatLxBI9gBaebbnrfifHhDYfgasaacH8akY=wiFfYdH8Gipec8Eeeu0xXdbba9frFj0=OqFfea0dXdd9vqai=hGuQ8kuc9pgc9s8qqaq=dirpe0xb9q8qiLsFr0=vr0=vr0dc8meaabaqaciaacaGaaeqabaqabeGadaaakeaacyGGsbGucqGGLbqzcqGH9aqpdaWcaaqaaGGaciab=f8aYnaaBaaaleaacqWGobGtcqWGmbataeqaaOGaemODay3aaSbaaSqaaiabd6eaojabdYeambqabaGccqWGebardaWgaaWcbaGaemOta4KaemitaWeabeaaaOqaaiab=X7aTnaaBaaaleaacqWGobGtcqWGmbataeqaaaaakiabg2da9maalaaabaGae8xWdi3aaSbaaSqaaiabd2eanjabdwfavbqabaGccqWG2bGDdaWgaaWcbaGaemyta0KaemyvaufabeaakiabdseaenaaBaaaleaacqWGnbqtcqWGvbqvaeqaaaGcbaGae8hVd02aaSbaaSqaaiabd2eanjabdwfavbqabaaaaaaa@515E@

where Re = characteristic Reynolds number, *ρ *= the fluid density, v = the fluid velocity, D = the tubing diameter, and, *μ *= the fluid viscosity; NL subscripts denote parameters of the normal volunteer study while MU denotes those of the Mock-up flow system.

The densities of both systems were assumed to be equal (*ρ*_NL _= *ρ*_MU_) while the diameter of the Mock-up system was twice that of the average normal volunteer vessel diameter (D_MU _= 2.0D_NL_). Furthermore, the viscosity of blood was three and a half times greater than the tap water in the Mock-up system (*μ*_NL _= 3.5 *μ*_MU_). These relationships allowed for the velocity of the Mock-up system to be determined relative to that of the normal volunteer study:

VMU=μMUDNLμNLDMUVNL=VNL7.0
 MathType@MTEF@5@5@+=feaafiart1ev1aaatCvAUfKttLearuWrP9MDH5MBPbIqV92AaeXatLxBI9gBaebbnrfifHhDYfgasaacH8akY=wiFfYdH8Gipec8Eeeu0xXdbba9frFj0=OqFfea0dXdd9vqai=hGuQ8kuc9pgc9s8qqaq=dirpe0xb9q8qiLsFr0=vr0=vr0dc8meaabaqaciaacaGaaeqabaqabeGadaaakeaacqWGwbGvdaWgaaWcbaGaemyta0Kaemyvaufabeaakiabg2da9maalaaabaacciGae8hVd02aaSbaaSqaaiabd2eanjabdwfavbqabaGccqWGebardaWgaaWcbaGaemOta4KaemitaWeabeaaaOqaaiab=X7aTnaaBaaaleaacqWGobGtcqWGmbataeqaaOGaemiraq0aaSbaaSqaaiabd2eanjabdwfavbqabaaaaOGaemOvay1aaSbaaSqaaiabd6eaojabdYeambqabaGccqGH9aqpdaWcaaqaaiabdAfawnaaBaaaleaacqWGobGtcqWGmbataeqaaaGcbaGaeG4naCJaeiOla4IaeGimaadaaaaa@4C69@

Next, the value of V_MU _was applied to the continuity equation as seen in Equation 3:

QMU=VMUAMU=VNL7.0π(2.0DNL2)2=0.449⋅VNLDNL  2
 MathType@MTEF@5@5@+=feaafiart1ev1aaatCvAUfKttLearuWrP9MDH5MBPbIqV92AaeXatLxBI9gBamXvP5wqSXMqHnxAJn0BKvguHDwzZbqegyvzYrwyUfgarqqtubsr4rNCHbGeaGqiA8vkIkVAFgIELiFeLkFeLk=iY=Hhbbf9v8qqaqFr0xc9pk0xbba9q8WqFfeaY=biLkVcLq=JHqVepeea0=as0db9vqpepesP0xe9Fve9Fve9GapdbaqaaeGacaGaaiaabeqaamqadiabaaGcbaGaemyuae1aaSbaaSqaaiabd2eanjabdwfavbqabaGccqGH9aqpcqWGwbGvdaWgaaWcbaGaemyta0KaemyvaufabeaakiabdgeabnaaBaaaleaacqWGnbqtcqWGvbqvaeqaaOGaeyypa0ZaaSaaaeaacqWGwbGvdaWgaaWcbaGaemOta4KaemitaWeabeaaaOqaaiabiEda3iabc6caUiabicdaWaaaiiGacqWFapaCdaqadaqaamaalaaabaGaeGOmaiJaeiOla4IaeGimaaJaemiraq0aaSbaaSqaaiabd6eaojabdYeambqabaaakeaacqaIYaGmaaaacaGLOaGaayzkaaWaaWbaaSqabeaacqaIYaGmaaGccqGH9aqpcqaIWaamcqGGUaGlcqaI0aancqaI0aancqaI5aqocqGHflY1cqWGwbGvdaWgaaWcbaGaemOta4KaemitaWeabeaakiabdseaenaaDaaaleaacqqGobGtcqqGmbataeaacqqGGaaicqqGGaaicqqGYaGmaaaaaa@6E16@

where Q_MU _= the volumetric flow rate in the Mock-up test system (L/min) and A_MU _= the cross-sectional area of the test section in the Mock-up system (cm^2^). This required that for V_NL _= 14.2 cm/s and D_NL _= 1.25 cm that Q_MU _= 0.599 L/min (corresponding to a mean Re = 146).

Testing of a given valve consisted of recording differential pressure and flow while adjusting the variable resistor in the recycle stream to obtain the desired flow to the test section. After the flow rate to the test section stabilized at the desired value for a few seconds, the pump was turned off and the flow allowed to recede under the force of gravity. The pump remained off until the level of the water reached an equilibrium point with the height of the fluid in Tower I. This protocol was repeated five times for each valve. Data recordings consisted of analog differential pressure and flow waveforms. Differential pressure was measured with a transducer (Model P305D, Validyne Engineering Corporation, Northridge, CA) whose ports were connected to the test section at a distance of 27 cm above and below the test valve to allow for placement of the device inside a view box. The transducer itself was set at a height of 23 cm above the reference tubing to match the elevation of the device. Flow was measured with a 16 mm inner diameter transonic flow probe connected to the system just proximal to the recycle stream. The signal was processed by a transonic flow meter (Model T108, Transonic Systems, Ithaca, NY). Ultrasonic reflectors for the flow transducer were not needed because of the mineral particles in the tap water. Both differential pressure and flow waveforms were relayed to a PC interface system (WINDAQ™, DATAQ^® ^Instruments Incorporated, Akron, OH) for recording and display. The waveforms for the Mock-up flow system were converted from a voltage signal to either their corresponding average differential pressure or flow values via linear calibration curves based on known data points. Through the acquisition system, data were obtained to the nearest 0.001 V with a temporal resolution of 1 ms at a sampling rate of 1000 Hz. Using the calibration equations, this corresponded to a resolution of 0.0345 mm Hg and 0.00264 L/min for differential pressure and flow rate, respectively.

Measured parameters for the 2:1 Mock-up system included mean flow rate, average differential pressure, valve closing time, reflux, and leakage. Each variable was defined by isolating a specific region in the time domain based on characteristic features of the differential pressure and flow waveforms. Mean flow rate and average differential pressure were time averaged over the period of one second before the constant flow pump was turned off. This provided the dynamic conditions that were acting on the valve as valve closure occurred. The time marker for the pump shut-off was defined as an initial sharp decrease in the flow waveform. The pump shut-off time was also used as the start marker for both the valve closing time and reflux parameters whereas the end marker was defined using the first rebound spike of the differential pressure waveform. Physically, the Valve Closing Time, **VCT**, was the time it took for the valve leaflets to first approximate one another after cessation of the forward driving pressure source. Correspondingly, Reflux Volume, VR
 MathType@MTEF@5@5@+=feaafiart1ev1aaatCvAUfKttLearuWrP9MDH5MBPbIqV92AaeXatLxBI9gBaebbnrfifHhDYfgasaacH8akY=wiFfYdH8Gipec8Eeeu0xXdbba9frFj0=OqFfea0dXdd9vqai=hGuQ8kuc9pgc9s8qqaq=dirpe0xb9q8qiLsFr0=vr0=vr0dc8meaabaqaciaacaGaaeqabaqabeGadaaakeaadaajbaqaaGqabiab=zfawbaadaWgaaWcbaGae8Nuaifabeaaaaa@2F65@, was defined as the volume of fluid that passed retrograde through the valve during the closing time period. The time interval over one second following the end marker was defined as the "1^st ^Leakage Interval" where the volume of fluid that leaked distally through the valve was the leakage. The same thing was done over the next second, which was the "2^nd ^Leakage Interval".

The only calculated parameter associated with the 2:1 Mock-up system was valve resistance, **R**, which was defined as the ratio of the average differential pressure to the mean flow rate. This value quantitatively described how much of a frictional effect the valve had on antegrade flow.

### b. 1:1 Physiologic Flow System

**Figure 2:**
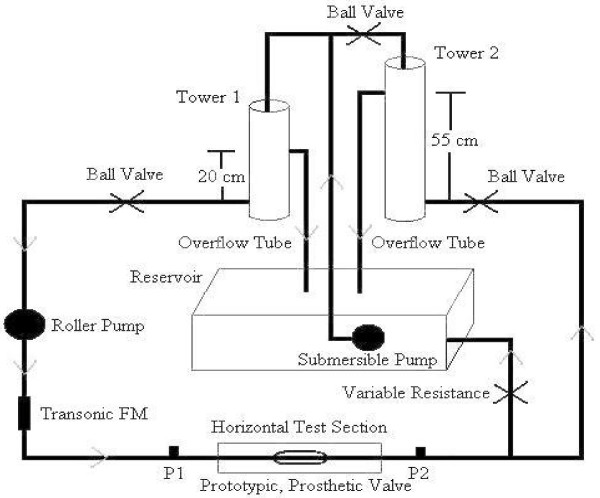
A schematic of the horizontal 1:1 Pulsatile Flow System.

The 1:1 Physiologic Flow System (Fig. 2) was intended to mimic the more complex conditions observed in the normal volunteer study. It combined steady and pulsatile flow components where steady flow was provided by a constant elevation (20 cm) reservoir (Tower I) and pulsatile flow was provided by a roller pump (Drake-Willock, Hemodialysis Systems, Oregon), which had been modified by the removal of one of the two rollers acting upon a compliant tube (bicycle inner tube). Cyclic flow output of the roller pump was superimposed onto the steady component by placing it in series with the output of Tower I and operating it at frequencies of 15 and 30 rpms to simulate ankle flexion and breathing conditions, respectively. A second constant elevation (55 cm) reservoir (Tower II) was also placed downstream of the valve. The purpose of Tower I was to simulate the residual arterial pressure entering the venous system while the purpose of Tower II was to simulate the hydrostatic backpressure induced in the standing position. The test section again contained a threaded adaptor similar to that of the Mock-up flow system, which facilitated valve installation and alignment while simultaneously preventing system leakage. The system was filled with a blood analog fluid composed of a mixture of tap water and 3.45% Dextran (SIGMA Chemical Company, St. Louis, MO). As with the 2:1 Mock-up system, data recordings consisted of analog differential pressure and flow waveforms with the only difference being that differential pressure was measured across ports separated by 31 cm on either side of the test valve, again to allow for mounting within a view box.

Testing of a given valve was performed by recording differential pressure and flow over each combination of body position (Supine or Standing) and dynamic condition (Breathing or Ankle Flexion) over forty cycles (Table 1). Procedurally, this was done by adjusting the steady flow rate to 0.35 L/min (Re = 170) using the variable resistance valve proximal to the test section while the pulsatile pump was off and the valve to Tower II closed. With the roller pump set at 15 rpm, the supine breathing (**SUBR**) simulation was created. After the first forty cycles, the standing breathing (**STBR**) condition was produced by opening the valve to Tower II. Halfway through the testing protocol, the roller pump was shut off, the steady flow rate set to 0.75 L/min (Re = 364), the roller pump reset to 30 rpm, and the valve to Tower II closed resulting in the supine ankle flexion (**SUAF**) simulation. Finally, after the 120^th ^cycle, standing ankle flexion (**STAF**) was created by opening the valve to Tower II. This protocol was repeated for each valve. Again, the waveforms for the physiological flow system were converted from voltage signals to either their corresponding average differential pressure or flow values via linear calibration curves with resolutions similar to those of the 2:1 Mock-up system.

**Table 1:** 1:1 Pulsatile Flow System Testing Protocol.

**Test Condition**	**Steady Flow rate (L/min)**	**Pulse Frequency (rpm)**	**Hydrostatic Backpressure**
**SUBR**	0.35	15	OFF
**SUAF**	0.75	30	OFF
**STBR**	0.35	15	ON
**STAF**	0.75	30	ON

Measured parameters obtained in the supine position included cycle period, mean flow rate, and average differential pressure. Valve closed time, reflux volume, and forward volume were the measured parameters for the simulated standing position. Again, each variable was defined by isolating a specific region in the time domain based on characteristic features of the differential pressure and flow waveforms. Extracting direct parameters of interest from the supine position data consisted of isolating a single flow cycle and running the statistics function of the data processing software. Doing so provided cycle period, mean flow rate, average differential pressure, and root mean square flow rate. Obtaining measured parameters from the standing position data was somewhat more complex in that it required dividing the flow waveform into two distinct temporal regions per cycle. The first region was the period in which flow was negative and was referred to as the "Valve Closed Time." The volume change during this period was considered the Reflux Volume, VR
 MathType@MTEF@5@5@+=feaafiart1ev1aaatCvAUfKttLearuWrP9MDH5MBPbIqV92AaeXatLxBI9gBaebbnrfifHhDYfgasaacH8akY=wiFfYdH8Gipec8Eeeu0xXdbba9frFj0=OqFfea0dXdd9vqai=hGuQ8kuc9pgc9s8qqaq=dirpe0xb9q8qiLsFr0=vr0=vr0dc8meaabaqaciaacaGaaeqabaqabeGadaaakeaadaajbaqaaGqabiab=zfawbaadaWgaaWcbaGae8Nuaifabeaaaaa@2F65@, while the volume change during the remainder of the cycle in which flow was positive was considered the Antegrade Volume, VA
 MathType@MTEF@5@5@+=feaafiart1ev1aaatCvAUfKttLearuWrP9MDH5MBPbIqV92AaeXatLxBI9gBaebbnrfifHhDYfgasaacH8akY=wiFfYdH8Gipec8Eeeu0xXdbba9frFj0=OqFfea0dXdd9vqai=hGuQ8kuc9pgc9s8qqaq=dirpe0xb9q8qiLsFr0=vr0=vr0dc8meaabaqaciaacaGaaeqabaqabeGadaaakeaadaajbaqaaGqabiab=zfawbaadaWgaaWcbaGae8xqaeeabeaaaaa@2F43@.

In the supine position, valve closure did not occur for either physiologic condition because the flow never reversed. Thus, the calculated parameters for this position evaluated the valve's influence on forward flow through the computation of Resistance, **R**, and Effective Orifice Area, **EOA**. Resistance was defined the same way as with the Mock-up flow system – the ratio of average differential pressure to mean flow rate and is only appropriate for describing steady flow systems. On the other hand, **EOA **(cm^2^) is a measure of the valve's orifice area based on a modified Venturi contraction and is a standard quantitative descriptor used for cardiac valves. It takes into account all of the losses associated with the valve under pulsatile flow conditions and is defined as:

EOA=QrmsCdΔP¯
 MathType@MTEF@5@5@+=feaafiart1ev1aaatCvAUfKttLearuWrP9MDH5MBPbIqV92AaeXatLxBI9gBaebbnrfifHhDYfgasaacH8akY=wiFfYdH8Gipec8Eeeu0xXdbba9frFj0=OqFfea0dXdd9vqai=hGuQ8kuc9pgc9s8qqaq=dirpe0xb9q8qiLsFr0=vr0=vr0dc8meaabaqaciaacaGaaeqabaqabeGadaaakeaacqWGfbqrcqWGpbWtcqWGbbqqcqGH9aqpdaWcaaqaaiabdgfarnaaBaaaleaacqWGYbGCcqWGTbqBcqWGZbWCaeqaaaGcbaGaem4qam0aaSbaaSqaaiabdsgaKbqabaGcdaGcaaqaamaanaaabaGaeuiLdqKaemiuaafaaaWcbeaaaaaaaa@3BF8@

where Q_rms _= the RMS flow rate in cm^3^/s, C_d _= the experimentally determined constant in cm⋅mmHg12⋅s−1
 MathType@MTEF@5@5@+=feaafiart1ev1aaatCvAUfKttLearuWrP9MDH5MBPbIqV92AaeXatLxBI9gBaebbnrfifHhDYfgasaacH8akY=wiFfYdH8Gipec8Eeeu0xXdbba9frFj0=OqFfea0dXdd9vqai=hGuQ8kuc9pgc9s8qqaq=dirpe0xb9q8qiLsFr0=vr0=vr0dc8meaabaqaciaacaGaaeqabaqabeGadaaakeaacqWGJbWycqWGTbqBcqGHflY1cqWGTbqBcqWGTbqBcqWGibascqWGNbWzdaahaaWcbeqaamaalaaabaGaeGymaedabaGaeGOmaidaaaaakiabgwSixlabdohaZnaaCaaaleqabaGaeyOeI0IaeGymaedaaaaa@3ECA@ and, ΔP¯
 MathType@MTEF@5@5@+=feaafiart1ev1aaatCvAUfKttLearuWrP9MDH5MBPbIqV92AaeXatLxBI9gBaebbnrfifHhDYfgasaacH8akY=wiFfYdH8Gipec8Eeeu0xXdbba9frFj0=OqFfea0dXdd9vqai=hGuQ8kuc9pgc9s8qqaq=dirpe0xb9q8qiLsFr0=vr0=vr0dc8meaabaqaciaacaGaaeqabaqabeGadaaakeaadaqdaaqaaiabfs5aejabdcfaqbaaaaa@2F4C@ = the average difference between systolic and diastolic pressures in mmHg.

While there is extensive experience in applying EOA to cardiac valve testing, it was felt that using the RMS flow rate and systolic and diastolic pressures was not appropriate for the venous system, particularly since more direct input measures were available. Thus, mean flow rate and differential pressure were used along with an empirical constant that was determined by measuring flow and differential pressure in the open tube (i.e. with 'No Device' in place), which corresponded to a known orifice area. Thus, the constant, C_d_, was solved for using Equation 4 for both the supine breathing and ankle flexion simulations.

Since valve closure and reflux only occurred in the simulated standing positions, all calculated parameters regarding reflux were obtained only for **STBR **and **STAF **conditions. The first of these was the Percent Reflux, **%Ref**, which was defined as the ratio of reflux volume to forward volume during one cycle so that it would be independent of small, yet distinct, deviations in the antegrade volume. The energy required to re-pump the reflux fluid through the valve, the Reflux Energy Loss, **REL **(J), was defined as:

REL=Fd=(ρVRg)(VRA)=ρgAVR 2
 MathType@MTEF@5@5@+=feaafiart1ev1aaatCvAUfKttLearuWrP9MDH5MBPbIqV92AaeXatLxBI9gBamXvP5wqSXMqHnxAJn0BKvguHDwzZbqegyvzYrwyUfgarqqtubsr4rNCHbGeaGqiA8vkIkVAFgIELiFeLkFeLk=iY=Hhbbf9v8qqaqFr0xc9pk0xbba9q8WqFfeaY=biLkVcLq=JHqVepeea0=as0db9vqpepesP0xe9Fve9Fve9GapdbaqaaeGacaGaaiaabeqaamqadiabaaGcbaGaemOuaiLaemyrauKaemitaWKaeyypa0JaemOrayKaemizaqMaeyypa0ZaaeWaaeaaiiGacqWFbpGCcqWGwbGvdaWgaaWcbaGaemOuaifabeaakiabdEgaNbGaayjkaiaawMcaamaabmaabaWaaSaaaeaacqWGwbGvdaWgaaWcbaGaemOuaifabeaaaOqaaiabdgeabbaaaiaawIcacaGLPaaacqGH9aqpdaWcaaqaaiab=f8aYjabdEgaNbqaaiabdgeabbaacqWGwbGvdaqhaaWcbaGaeeOuaifabaGaeeiiaaIaeeOmaidaaaaa@5AAB@

where F = force (N), d = distance (m), *ρ *= water density (kg/m^3^), VR
 MathType@MTEF@5@5@+=feaafiart1ev1aaatCvAUfKttLearuWrP9MDH5MBPbIqV92AaeXatLxBI9gBaebbnrfifHhDYfgasaacH8akY=wiFfYdH8Gipec8Eeeu0xXdbba9frFj0=OqFfea0dXdd9vqai=hGuQ8kuc9pgc9s8qqaq=dirpe0xb9q8qiLsFr0=vr0=vr0dc8meaabaqaciaacaGaaeqabaqabeGadaaakeaadaajbaqaaGqabiab=zfawbaadaWgaaWcbaGae8Nuaifabeaaaaa@2F65@ = Reflux volume (m^3^), g = the gravitational constant (m/s^2^), and, A = the cross sectional area of the test section (m^2^).

Another term, the Antegrade Energy, **AE**, was defined similar to **REL**, where **AE **(J) quantified the energy produced by the pump in propelling the fluid proximally:

AE=ρgAVA 2
 MathType@MTEF@5@5@+=feaafiart1ev1aaatCvAUfKttLearuWrP9MDH5MBPbIqV92AaeXatLxBI9gBamXvP5wqSXMqHnxAJn0BKvguHDwzZbqegyvzYrwyUfgarqqtubsr4rNCHbGeaGqiA8vkIkVAFgIELiFeLkFeLk=iY=Hhbbf9v8qqaqFr0xc9pk0xbba9q8WqFfeaY=biLkVcLq=JHqVepeea0=as0db9vqpepesP0xe9Fve9Fve9GapdbaqaaeGacaGaaiaabeqaamqadiabaaGcbaGaemyqaeKaemyrauKaeyypa0ZaaSaaaeaaiiGacqWFbpGCcqWGNbWzaeaacqWGbbqqaaGaemOvay1aa0baaSqaaiabbgeabbqaaiabbccaGiabbkdaYaaaaaa@4865@

where VA
 MathType@MTEF@5@5@+=feaafiart1ev1aaatCvAUfKttLearuWrP9MDH5MBPbIqV92AaeXatLxBI9gBaebbnrfifHhDYfgasaacH8akY=wiFfYdH8Gipec8Eeeu0xXdbba9frFj0=OqFfea0dXdd9vqai=hGuQ8kuc9pgc9s8qqaq=dirpe0xb9q8qiLsFr0=vr0=vr0dc8meaabaqaciaacaGaaeqabaqabeGadaaakeaadaajbaqaaGqabiab=zfawbaadaWgaaWcbaGae8xqaeeabeaaaaa@2F43@ = the Antegrade volume (m^3^).

Finally, the **REL **was compared to the **AE **to provide the Energy Retention, ***η***:

η=1−RELAE
 MathType@MTEF@5@5@+=feaafiart1ev1aaatCvAUfKttLearuWrP9MDH5MBPbIqV92AaeXatLxBI9gBaebbnrfifHhDYfgasaacH8akY=wiFfYdH8Gipec8Eeeu0xXdbba9frFj0=OqFfea0dXdd9vqai=hGuQ8kuc9pgc9s8qqaq=dirpe0xb9q8qiLsFr0=vr0=vr0dc8meaabaqaciaacaGaaeqabaqabeGadaaakeaaiiGacqWF3oaAcqGH9aqpcqaIXaqmcqGHsisldaWcaaqaaiabdkfasjabdweafjabdYeambqaaiabdgeabjabdweafbaaaaa@36D1@

Statistical analysis for the Mock-up data consisted of a one-way analysis of variance (ANOVA) at a significance level of 0.05 performed on mean flow rate, which was the controlled variable, and a linear regression was performed between Valve Closure Time and Reflux. For the physiologic data, a series of one-way ANOVA tests were run at a significance level of 0.05 on the variables that were most relevant to valve selection. These parameters included average **ΔP**, **R**, **EOA**, VR
 MathType@MTEF@5@5@+=feaafiart1ev1aaatCvAUfKttLearuWrP9MDH5MBPbIqV92AaeXatLxBI9gBaebbnrfifHhDYfgasaacH8akY=wiFfYdH8Gipec8Eeeu0xXdbba9frFj0=OqFfea0dXdd9vqai=hGuQ8kuc9pgc9s8qqaq=dirpe0xb9q8qiLsFr0=vr0=vr0dc8meaabaqaciaacaGaaeqabaqabeGadaaakeaadaajbaqaaGqabiab=zfawbaadaWgaaWcbaGae8Nuaifabeaaaaa@2F65@, **%Ref**, **REL**, and ***η***. The analysis was performed for both dynamic conditions in which the respective parameter was measured. When a significant difference existed, grouping was provided by the SNK procedure and was performed by a computer program (SAS, SAS Institute Incorporated, Cary, NC). To determine the repeatability of the system, one prototype valve was tested, removed and then re-inserted into the system for repeat testing. Those results were then evaluated to test for any statistical differences.

## Results

Results of the normal volunteer study were extremely valuable in defining both typical dimensions and anatomy of veins in the proximal lower extremity, but also in quantifying the velocities, flow rates, and cycle periods under various postural and physiologic conditions. Specifically, over the 11 subjects, the common femoral vein diameter was 1.04 ± 0.14 cm and peak flow rates (derived from peak velocities) were 0.6 L/min, 1.34 L/min, 0.78 L/min and 1.6 L/min for the cases **SUBR**, **SUAF**, **STBR **and **STAF**, respectively. Based on these data, a diameter of 1.25 cm was chosen for the prosthetic valve design to allow for some distention of the vein upon insertion. Representative values for peak flow rate under breathing and ankle flexion conditions were taken (0.69 L/min and 1.5 L/min, respectively) as the average values measured in the two postural positions (Supine and Standing), since the breathing and ankle flexion movements acted as the primary driving forces in each case. These peak values were then obtained in the 1:1 Pulsatile Flow System by setting the steady flow component to 50% of those values (0.35 L/min and 0.75 L/min, respectively) and allowing the roller pump to superimpose an additional unsteady component. Pumping frequencies were based upon typical breathing (~15 breaths/min = 15 rpm, or 0.25 Hz) and walking (~30 steps/limb/min = 30 rpm, or 0.5 Hz) cycles.

**Figure 3:**
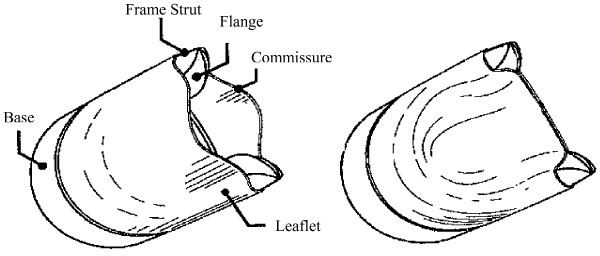
Prototype design of venous valve in the Open (left) and Closed (right) positions consisting of a solid circular base, frame struts with uniquely shaped flanges, and a flexible leaflet material.

To illustrate the overall testing procedure, a preliminary vein valve design was developed (Fig. 3). This device has several novel features [18] and was conceived in order to allow for a normally open device that fully closes upon application of backpressure. A 2:1 scale device was built using SLA fabrication techniques and incorporating the threaded extension as described earlier. The device frame was then wrapped with a 5 mil (127 *μ*m) thick sheet of Biospan^® ^(Polymer Technology Group, Berkeley, CA) to serve as leaflets. These were cut to a shape such that the closed leaflet edges contacted along a straight line across the valve lumen.

### a. 2:1 Mock-Up System

**Figure 4:**
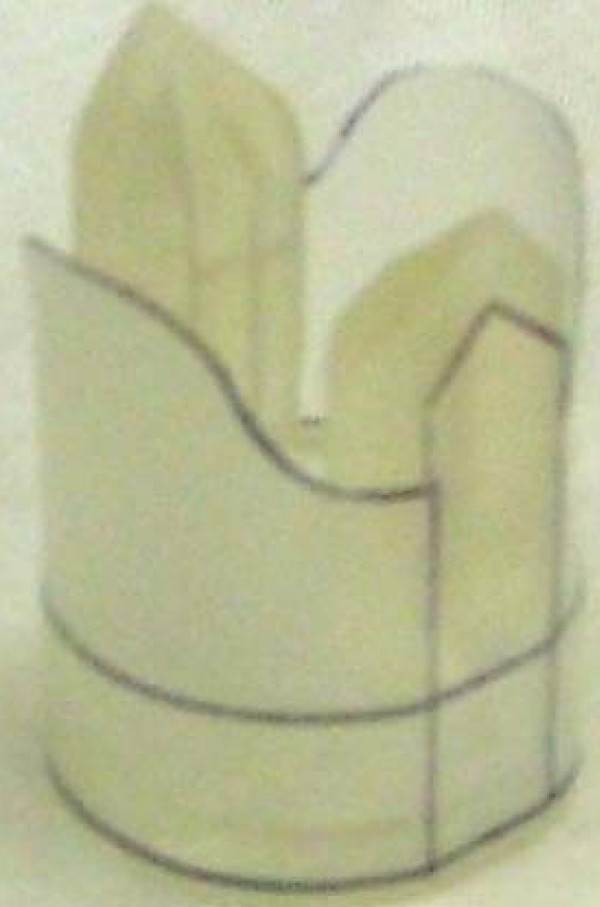
Actual 2:1 scale device with optimized frame and shaped Biospan^® ^leaflet. A threaded extension from the base (not shown) screwed into the test section fixture to improve alignment and prevent leakage.

The prototype device was then installed in the 2:1 Mock-up system and evaluated as described earlier. Initial observations showed that the leaflet had a natural tendency to fold inwardly along a nearly diagonal line between the tip of the flange and a point halfway between the two flanges along the base. Additional observations also showed that, for the leaflets to close quickly and reliably, they needed to have some initial inward deflection. These two issues were resolved by modifications to the side of the frames and means of attachment of the leaflet to the base. A threaded extension was also added at this point to improve axial alignment of the device and to reduce leakage around connectors (Fig. 4).

A set of these devices was fabricated and a series of tests were then made on these designs in the 2:1 Mock-up System. Key results for the prototype device were that it had a forward steady flow pressure drop of 1.25 ± 0.153 mmHg and a resistance of 1.92 ± 0.578 mmHg*min/L. Under sudden hydrostatic backpressure conditions, the valve required 1.38 ± 0.0447 s to close and allowed 19.9 ± 4.40 cm^3 ^of reflux followed by 4.36 ± 1.48 cm^3 ^and 1.23 ± 1.02 cm^3 ^of leakage during the 1^st ^and 2^nd ^one second post-closure intervals, respectively.

### b. 1:1 Physiologic Flow System

After completion of tests on a series of such devices, 1:1 prototypes were similarly constructed and evaluated in the pulsatile flow system to see what differences dimensional scale and test conditions would make. Results of these tests are shown for the 'No Device' case and one valve prototype under supine and standing conditions in Tables 2 and 3, respectively. The purpose of the 'No Device' case was: 1) to quantify losses in the system (i.e. tubing, connectors, etc.) which were not related to the test device itself, and, 2) to serve as a baseline for worst case values that would be seen with a completely incompetent valve. [Note: For the EOA calculations, values for C_d _under the 'No Device' case were 2.32 ± 0.03 cm⋅mmHg12⋅s−1
 MathType@MTEF@5@5@+=feaafiart1ev1aaatCvAUfKttLearuWrP9MDH5MBPbIqV92AaeXatLxBI9gBaebbnrfifHhDYfgasaacH8akY=wiFfYdH8Gipec8Eeeu0xXdbba9frFj0=OqFfea0dXdd9vqai=hGuQ8kuc9pgc9s8qqaq=dirpe0xb9q8qiLsFr0=vr0=vr0dc8meaabaqaciaacaGaaeqabaqabeGadaaakeaacqWGJbWycqWGTbqBcqGHflY1cqWGTbqBcqWGTbqBcqWGibascqWGNbWzdaahaaWcbeqaamaalaaabaGaeGymaedabaGaeGOmaidaaaaakiabgwSixlabdohaZnaaCaaaleqabaGaeyOeI0IaeGymaedaaaaa@3ECA@ and 6.38 ± 0.142 cm⋅mmHg12⋅s−1
 MathType@MTEF@5@5@+=feaafiart1ev1aaatCvAUfKttLearuWrP9MDH5MBPbIqV92AaeXatLxBI9gBaebbnrfifHhDYfgasaacH8akY=wiFfYdH8Gipec8Eeeu0xXdbba9frFj0=OqFfea0dXdd9vqai=hGuQ8kuc9pgc9s8qqaq=dirpe0xb9q8qiLsFr0=vr0=vr0dc8meaabaqaciaacaGaaeqabaqabeGadaaakeaacqWGJbWycqWGTbqBcqGHflY1cqWGTbqBcqWGTbqBcqWGibascqWGNbWzdaahaaWcbeqaamaalaaabaGaeGymaedabaGaeGOmaidaaaaakiabgwSixlabdohaZnaaCaaaleqabaGaeyOeI0IaeGymaedaaaaa@3ECA@ for the **SUBR **and **SUAF **conditions, respectively.] Finally, the results from repeatability testing of these variables over two runs on the same prototype valve design under supine and standing conditions are shown in Tables 4 and 5, respectively.

**Table 2:** 1:1 Pulsatile Flow Results for Prototype vs. 'No Device' in Supine Position.

**Test Condition**	**Device**	**ΔP (mmHg)**	R (mmHg*min/L)	**EOA (cm^2^)**
**SUBR**	None	13.3 ± 0.0272	21.3 ± 0.271	1.23*
	Prototype	14.6 ± 0.0012	23.5 ± 0.0338	1.12 ± 0.0166
	Change	1.33	2.23	0.11
**SUAF**	None	7.97 ± 0.0935	6.00 ± 0.163	1.23*
	Prototype	8.81 ± 0.157	7.37 ± 0.110	1.09 ± 0.0111
	Change	1.84	1.37	0.14

* Calculated value based on tube dimensions

**Table 3:** 1:1 Pulsatile Flow Results for Prototype vs. 'No Device' in Standing Position.

**Test Condition**	**Device**	VR MathType@MTEF@5@5@+=feaafiart1ev1aaatCvAUfKttLearuWrP9MDH5MBPbIqV92AaeXatLxBI9gBaebbnrfifHhDYfgasaacH8akY=wiFfYdH8Gipec8Eeeu0xXdbba9frFj0=OqFfea0dXdd9vqai=hGuQ8kuc9pgc9s8qqaq=dirpe0xb9q8qiLsFr0=vr0=vr0dc8meaabaqaciaacaGaaeqabaqabeGadaaakeaadaajbaqaaGqabiab=zfawbaadaWgaaWcbaGae8Nuaifabeaaaaa@2F65@**(cm^3^)**	**%Ref (%)**	**REL (mJ)**	***η *(%)**
**STBR**	None	59.6 ± 0.830	205 ± 5.37	283 ± 7.93	-319 ± 22.1
	Prototype	5.83 ± 0.688	18.1 ± 2.12	2.73 ± 0.654	36.7 ± 0.785
**STAF**	None	7.35 ± 0.28	24.6 ± 0.859	4.30 ± 0.333	93.9 ± 0.429
	Prototype	2.21 ± 0.978	7.10 ± 3.27	0.451 ± 0.411	99.4 ± 0.562

**Table 4:** 1:1 Pulsatile Flow Results for Repeat Measurements in Supine Position.

**Test Condition**	**Test Run**	**ΔP^1 ^(mmHg)**	R^1 ^(mmHg*min/L)	**EOA (cm^2^)**
**SUBR**	1^st^	1.33 ± 0.0012	2.23 ± 0.0338	1.12 ± 0.0166
	2^nd^	1.41 ± 0.0221*	2.29 ± 0.0379*	1.16 ± 0.015*
	Change	0.08	0.06	0.04
**SUAF**	1^st^	1.84 ± 0.151	1.37 ± 0.110	1.09 ± 0.0111
	2^nd^	2.33 ± 0.0926*	1.73 ± 0.0889*	1.08 ± 0.0215
	Change	0.49	0.36	0.01

^1 ^Relative to 'No Device' value

* p < 0.05 vs. 1^st ^Test Run

**Table 5:** 1:1 Pulsatile Flow Results for Repeat Measurements in Standing Position.

**Test Condition**	**Test Run**	VR MathType@MTEF@5@5@+=feaafiart1ev1aaatCvAUfKttLearuWrP9MDH5MBPbIqV92AaeXatLxBI9gBaebbnrfifHhDYfgasaacH8akY=wiFfYdH8Gipec8Eeeu0xXdbba9frFj0=OqFfea0dXdd9vqai=hGuQ8kuc9pgc9s8qqaq=dirpe0xb9q8qiLsFr0=vr0=vr0dc8meaabaqaciaacaGaaeqabaqabeGadaaakeaadaajbaqaaGqabiab=zfawbaadaWgaaWcbaGae8Nuaifabeaaaaa@2F65@**(cm^3^)**	**%Ref (%)**	**REL (mJ)**	***η *(%)**
**STBR**	1^st^	5.83 ± 0.688	18.1± 2.12	2.73 ± 0.654	36.7 ± 0.785
	2^nd^	3.99 ± 0.537*	12.7 ± 2.07*	1.29 ± 0.371*	38.4 ± 0.52*
	^Change^	1.84	5.4	1.44	1.7
**STAF**	1^st^	2.21 ± 0.978	7.10 ± 3.27	0.451 ± 0.411	99.4 ± 0.562
	2^nd^	1.55 ± 1.74	4.75 ± 5.27	0.384 ± 0.375	99.6 ± 0.443
	Change	0.66	2.35	0.067	0.2

* p < 0.05 vs. 1^st ^Test Run

## Discussion

### a) 2:1 Mock-Up System

From a procedural standpoint, the Mock-up flow system proved to be an essential element in the development of second-generation optimized valve designs. The 2:1 scale and the steady flow component allowed for detailed observations to be made over the course of an extended time period, which would have been much more difficult to make on the 1:1 Physiologic Flow System alone. Similarly, the vertical column arrangement provided the ability to study the valve when it was subjected to the most extreme clinical condition. The Mock-up system was particularly valuable in providing information about frame-leaflet interactions and how the leaflet collapsed under a uniform reversing pressure. These capabilities led to observations that initiated the incorporation of features that were essential to the dynamic performance of the leaflets. Thus, it was concluded that the Mock-up was a necessary intermediate step in the design and development of the optimally designed valves.

Although dynamic similarity was maintained, the data obtained from the Mock-up flow system were not used to select the best performing second-generation optimized design. However, these results were able to indicate that the test device did have low forward resistance and reasonably low reflux. [Note: It was at this point in the design process that consideration was given to: 1) devise outcome parameters which were more specific to vein valve operation, and, 2) include recordings of tests with 'No Device' installed so that direct comparisons could be made of the prototype as a measure of relative effectiveness versus a totally incompetent valve.]

### b) 1:1 Physiologic Flow System

The 1:1 Physiologic Flow System was valuable in simulating four realistic postural and physiologic conditions (**SUBR**, **SUAF**, **STBR**, and **STAF) **for vein valve testing, which provided data to evaluate the second-generation, optimally designed valves. The physiologic system was relevant to the dynamic conditions of the veins in the leg, which were necessary for a full evaluation of the different valves of the second generation, optimized design.

More specifically, by reducing the prosthetic devices to life-size (i.e. 1:1 scale) models, it was possible to obtain more realistic results and to confirm whether the devices would operate as well under physiologic conditions. However, this scale reduction also magnified some of the limitations in function of the device such as increased flow resistance and greater reflux due to decreased ability of the leaflet material to conform to the strut shapes. The smaller size also reduced precision of the fabrication process and, therefore, increased the relative impact of any incurred defects. Furthermore, by testing in a pulsatile flow system, it was possible to evaluate the effects of larger inertial forces and cyclical flexion on the device. This was important because opening and closing of the valve is strongly dependent upon the peak dynamic forces of the fluid and because of possible deformation or failure responses of the device after repeated operation. It was observed that the shorter durations of the opening and closing phases produced by the oscillatory flow conditions resulted in some designs not being able to close quickly enough and that the leaflets tended to achieve a more repeatable mode of operation after several cycles.

Unfortunately, there are virtually no comparable data from other prosthetic vein valves and it is unrealistic to compare these values to those of prosthetic cardiac valves due to large differences in test conditions – i.e. higher operating pressures (~2×), flow rates (~10×) and shorter, more abrupt cycles. As mentioned earlier, however, several outcome parameters were either modified or devised during this procedure in order to better quantify certain important device characteristics. For example, **EOA **was included because of its widespread use in evaluating cardiac valves. However, the standard definition of **EOA **is based on input data readily available to clinicians, i.e. systolic and diastolic pressures and root-mean-square flow, rather than more direct measures of pressure difference and flow rate *per se*. Furthermore, while the reflux volume is important, the question arose as to "how much reflux is too much?" Since it was difficult to estimate the amount of excess volume that the lower limb could tolerate over a given period of time, an alternate approach to this issue was taken by simply looking at the energy 'cost' of re-pumping the reflux flow. This, then, led to the Reflux Energy Loss (**REL**) parameter, which measured the work needed to lift the reflux volume of fluid against gravity the equivalent height of one point just distal to one point just proximal to the valve. Finally, collection of data with 'No Device' installed proved very useful for making comparisons of a given device (regardless of its overall ranking) against the worst possible case to determine the improvement it provided versus a totally defective valve (i.e. the clinical equivalent of CVI). These measurements also helped to establish the full-scale range of a given parameter.

Ultimately, the pulsatile flow system provided all of the data used for valve selection. In particular, the **SUBR **and **SUAF **conditions allowed for the evaluation of valves in terms of **R **and **EOA **while the **STBR **and **STAF **simulations characterized valves according to VR
 MathType@MTEF@5@5@+=feaafiart1ev1aaatCvAUfKttLearuWrP9MDH5MBPbIqV92AaeXatLxBI9gBaebbnrfifHhDYfgasaacH8akY=wiFfYdH8Gipec8Eeeu0xXdbba9frFj0=OqFfea0dXdd9vqai=hGuQ8kuc9pgc9s8qqaq=dirpe0xb9q8qiLsFr0=vr0=vr0dc8meaabaqaciaacaGaaeqabaqabeGadaaakeaadaajbaqaaGqabiab=zfawbaadaWgaaWcbaGae8Nuaifabeaaaaa@2F65@, **%Ref**, **REL**, and ***η***. Both supine simulations were found to produce fairly realistic conditions but neither induced valve closure. This was consistent with observations made earlier during the normal volunteer study and focused the value of these tests on resistance-based, rather than reflux-based, parameters. The results obtained under the **SUBR **condition were considered particularly relevant since this case had a longer cycle period (4 s versus 2 s) and, thus, was more 'quasi-steady'. Reflux, on the other hand, was observed with both standing positions. However, the **STAF **simulation did not allow as much reflux as the **STBR **condition because of the higher frequency of the roller pump, which acted as an effective valve and, thus, limited reflux. For example, in the 'No Device' case, only 24.6% reflux was observed under the **STAF **condition as compared to 205% reflux in the **STBR **condition. Therefore, it was concluded that test valves would not be exposed to reversing flows under the **STAF **condition, which are large enough to distinguish between valve designs. The finding that **%Ref **exceeded 100% and ***η ***exceeded -300% for **STBR **underscores the fact that back flow in the veins is not primarily dependent upon the amount of forward flow *per se *but rather, upon the backpressure acting on the valve. In other words, a successful vein valve should first be able to prevent backflow of blood from the entire proximal vein and then, secondly, it should limit backflow beyond the point where the net forward flow is less than that required to maintain normal flow through the vessel.

Repeatability testing showed that, for a representative device, there were some statistically different values in the measured and computed variables between runs. These differences existed for all parameters under simulated 'breathing' conditions (i.e. **SUBR **and **STBR**) but only 2 of 7 parameters (**ΔP **and **R**) under simulated 'ankle flexion' conditions (i.e. **SUAF **and **STAF**). While these differences will limit the test system's ability to distinguish one valve design from another to some extent, most of the variability is relatively small compared to the 'No Device' case [Tables 2 and 3], and thus, should accurately reflect the improvement expected compared to a completely defective valve.

Finally, it is important to note that neither of these *in vitro *test systems used collapsible tubing in the test section as some earlier studies had done (15–17). While this feature would clearly have altered the results obtained, and especially the reflux flow data, it was felt that during the initial design testing phase, it was best to use a system which would evaluate the capabilities of the various devices themselves independent of other factors. Based on those results, then, appropriate improvements could be incorporated and/or final selections made between competing designs. Once optimized, the final design could then be re-tested with the addition of collapsible tubing to determine the physiologic response of the prototypic valve + vein combination.

## Conclusion

From these results, it was concluded that the initial test device produced only a small increases in **ΔP **(<0.15 mmHg) and **R **(<0.25 mmHg*min/L) and a small decrease in **EOA **(<8%) relative to 'No Device'. Furthermore, it allowed <2% of VR
 MathType@MTEF@5@5@+=feaafiart1ev1aaatCvAUfKttLearuWrP9MDH5MBPbIqV92AaeXatLxBI9gBaebbnrfifHhDYfgasaacH8akY=wiFfYdH8Gipec8Eeeu0xXdbba9frFj0=OqFfea0dXdd9vqai=hGuQ8kuc9pgc9s8qqaq=dirpe0xb9q8qiLsFr0=vr0=vr0dc8meaabaqaciaacaGaaeqabaqabeGadaaakeaadaajbaqaaGqabiab=zfawbaadaWgaaWcbaGae8Nuaifabeaaaaa@2F65@ and <6% of the **REL **observed with 'No Device'. Thus, the overall test systems and procedures used proved quite valuable in carrying out this phase of the design process. Further studies are now planned to evaluate biocompatibility concerns such as thrombogenicity and intimal hyperplasia of these devices *in vivo*.

## Competing interests

The author(s) declare that they have no competing interests.

## Authors' contributions

SER designed the normal volunteer study and participated in the data analysis, conceived of the overall design and construction of the *in vitro *model system, specified the testing protocol and definition of measured variables, and participated in the data collection. MTO prepared design drawings of new devices, assisted in test data collection, and performed the data analysis. SP assisted in the normal volunteer study, performed the data analysis and assisted in construction of the *in vitro *model system. Each author has read and approved the final version of this manuscript.
